# Decoding episodic autobiographical memory in naturalistic virtual reality

**DOI:** 10.1038/s41598-024-76944-3

**Published:** 2024-10-27

**Authors:** Diane Lenormand, Inès Mentec, Alexandre Gaston-Bellegarde, Eric Orriols, Pascale Piolino

**Affiliations:** 1 Laboratoire Mémoire, Cerveau & Cognition, Institut de Psychologie, Université Paris Cité, Paris, LMC2 UR 7536 France; 2 Unité de recherche Conscience, Cognition et Computation, Faculté de Psychologie, Sciences de l’Éducation et Logopédie, Université Libre de Bruxelles, Bruxelles, Belgique

**Keywords:** Episodic autobiographical memory, Episodic memory, Virtual reality, Prediction, Emotion, Self, Human behaviour, Learning and memory, Consolidation

## Abstract

Episodic autobiographical memory (EAM) is a long-term memory system of personally experienced events with their context – *what*, *where*, *when* – and subjective elements, e.g., emotions, thoughts, or self-reference. EAM formation has rarely been studied in a controlled, real-life-like paradigm, and there is no predictive model of long-term retrieval from self-rated subjective experience at encoding. The present longitudinal study, with three surprise free recall memory tests immediately, one-week and one-month after encoding, investigated incidental encoding of EAM in an immersive virtual environment where 30 participants either interacted with or observed specific events of varying emotional valences with simultaneous physiological recordings. The predictive analyses highlight the temporal dynamics of the predictors of EAM from subjective ratings at encoding: common characteristics related to sense of remembering and infrequency of real-life encounter of the event were identified over time, but different variables become relevant at different time points, such as the emotion and mental imagery or prospective aspects. This dynamic and time-dependent role of memory predictors challenges traditional views of a uniform influence of encoding factors over time. Current evidence for the multiphasic nature of memory formation points to the role of different mechanisms at play during encoding but also consolidation and subsequent retrieval.

**Supplementary Information:**

The online version contains supplementary material available at 10.1038/s41598-024-76944-3.

## Introduction

The experimental exploration of episodic memory (EM) entails some challenges inherent to the very nature of EM. Tulving^1^ defines EM as a long-term memory system that allows us to encode, store, and retrieve personally experienced events. Therefore, EM comprises the encoding and retrieval of an event and the precise context in which it was encountered: *what* happened, *when*, *where*, and *how *it happened. This contextual information is usually associated with phenomenological details, such as emotions, thoughts, or self-reference; sensory-motor information, such as visual, auditory, or tactile details; and idiosyncratic aspects of the event. The association of all this information makes for a rich and detailed memory that can be subjectively revisited through mental time travel or autonoetic consciousness^2^. Specific, personal, real-life events that tend to be included in the construction of one’s self and one’s life story presuppose long retention intervals and constitute the episodic parts of autobiographical memory (episodic autobiographical memory, EAM)^3,4^. With a crucial role of oneself as a participant, EAM ensures the continuity of our identity through time^5^.

The traditional exploration of the formation of EM and EAM in a laboratory setting usually involves tasks that are loosely related to their real-world counterparts. This is the case of verbal paradigms, in which participants have to, intentionally or incidentally, learn a list of words and are then tested on their memory of that list. Such paradigms may involve other factors of interest, such as the emotional intensity or valence or a self-reference versus other-reference encoding (see for instance McKenzie and Humphreys^6^, Sharot and Phelps^7^, Pereira, et al.^8^, Moses-Payne, et al^9^.). Efforts have been made to develop more ecological experimental paradigms involving real-life activities such as a museum tour^10–12^and staged experiences^13,14^, everyday events recorded with wearable cameras^15–18^, traditional^19,20^ and video^21^ diaries, voice recordings^22,23^, 360° videos^24^, the use of a cell phone to encode new information^25^. Such paradigms tend to be more daily-life-like and mimic event memory properties that can potentially lead to EAM. They have begun to successfully circumvent the issues of person-dependent (active role of participants, embodiment, and sense of agency) and situation-dependent factors (contextualization of the tasks), two traditional limitations of laboratory settings^26^. Naturalistic approaches have provided new insights into memory with embodied, social, emotional, and realistic inputs^27^. They have established fundamental results regarding, for instance, the decrease of event memory performance and the increase of false memories over time^19^, the low percentage of retrieval of highly ecological details but their very high accuracy despite age and time declines^13^, and are leading the way towards potential applications in rehabilitation and aging^24^. Furthermore, these studies have found compelling results about EAM patterns of activation during retrieval. The prefrontal cortex and medial temporal region^22^, and the hippocampus and parahippocampal cortex^23^notably play a role in this retrieval; the medial parietal cortex is involved in the memory of dynamic real-life events^15^ and for autobiographical memories^21^. The implication of the medial temporal region is reflected in studies with memory-impaired patients with medial temporal lobe damage^14^, whose account of naturalistic events resembles that of healthy participants tested after more than two years. Despite an age-related deficit^18^, both young and older adults benefit from the presentation of sequential cues from the events during recognition^16,17^As for reactivation- and rehearsal-induced plasticity, a coherent reactivation with one’s experience improves subsequent recognition^11^, with a lesser effect in aging^10^; rehearsal also increases reported vividness^23^. Therefore, several variables of interest for EAM can be highlighted, in line with previous studies on long-lasting autobiographical memories (see for instance Piolino, et al.^28^ and Viard, et al.^29^): mental imagery, emotion, self-relevance and the sense of remembering, mental repetition and repetition with others (internal and external). Moreover, additional factors related specifically to encoding can be added to this list, for instance, the extent to which one expects to remember an event in the future, and how surprising or rare in real-life an event is^30–32^. Naturalistic paradigms have thus allowed the exploration of EAM with many different variables of interest, in a context closer to the one expected to happen in real life, following Neisser’s ecological approach^33^. However, they mostly still lack items that both include the same influence factors as real-life events and allow strict control by the experimenters.

The emergence and subsequent development of virtual reality (VR) has quickly demonstrated that fully ecological and immersive experimental paradigms are possible in a controlled laboratory setting^34^. VR is a 3D technology that allows a user to act in a digital environment as if it were the real world^35^. A virtual environment (VE) is experienced through computer-generated sensory stimuli and real-time user’s immersion and interaction that give a subjective sense of presence. Near-realistic VEs can be built to include multisensory (visual, auditory, even motor with joysticks or tactile with gloves) daily-life-like events and a chosen background, for instance museums^36^, parks^37^, towns^38,39^ etc., with different properties (affective, social…) that can be controlled by the experimenter^40,41^. In addition with the potential of being both immersive and interactive^41,42^, VEs allow the user to ‘behave and feel as if [they] were in the virtual world’, that is, to feel present in the VE^43^. As a result, VR is used in clinical settings, as well as in cognitive neuroscience^40^. More precisely, VR has been validated as an ecological assessment tool of EM in children, adolescents, young adults^44^, and normal and pathological aging^42,45,46^. VEs present indeed several advantages to study EAM: they allow more ecological paradigms, while preserving complete control from the experimenters, thus getting closer to the real-life encoding conditions where several factors interact. Burgess, et al.^39^ notably investigated EM activations for memory binding for contextual and spatial properties through a VE in which participants are shown objects by characters; more recently, Rey, et al.^47^ shed light on multisensory binding with boxes in different places in the VE, with either a music, an odour, or the picture of a face. In VR, active interaction with the VE, route planning and free navigation also benefit memory^38,48^, when the type of navigation does not require too much from the user’s executive abilities^49^. With an active encoding (real-life walking during VR), objects closer in a spatial-temporal context are better recognised^50^. However, the levels of immersion and interaction still vary in the VR settings in which EM has been studied^51^. Electroencephalography (EEG) has already been used, after the use of VR during retrieval, to show that VR promotes memory retrieval conditions and effort closer to real-life than computer-based laboratory settings, with a presumed lower visual effort during retrieval for VR conditions compared to a traditional setting^52^. Naturalistic EM, as explored simultaneously with VR and fMRI, likewise involves well-known brain regions for EM activation and additional regions related to the narrative and bodily self^53^.

The immersive property of VR allows users to express behavioural and physiological responses similar to those in real-life situations. For instance, the electrodermal activity (EDA) is the same in VR as in real-life for identical expertise level and cognitive load^54^. Furthermore, VR notably allows to elicit different emotions (e.g., joy, sadness, anger, boredom, anxiety, see Felnhofer, et al.^55^), in a similar way to real-life^36^. This emotion elicitation can go as far as emotion regulation^56^. Coincidentally, emotion is well-known to influence EM and EAM^57^. Emotions, separated in two orthogonal axes with valence and intensity^58,59^, modulate and moderate how memories are formed and retrieved^60,61^. Some consider intensity to have a greater effect on memory, highlighting the focal enhancement of arousal^62,63^, while others defend a more prominent role of valence: a negative valence seems to improve long-term binding^63,64^. More particularly in VR, emotions and EM have scarcely been explored concurrently. Zlomuzica, et al.^65^ showed that an induced anxious state leads to decreased memory performance. More recently, Billet, et al.^66^ investigated the link between subjective (‘person-related’, e.g. thoughts, emotions) and objective (i.e. contextual) elements and age using eight actions performed in VR and subsequently recalled: they found no age-related effect on the subjective part of recollection. The authors hypothesised that this could be related to low emotional engagement during the VR tasks; however, subjective recollection was associated with a higher reliving judgement. The input of EEG and heart-rate variability from electrocardiography (ECG) signals can satisfyingly predict both the subjective valence and intensity of an event in VR^67^. Moreover, outside of VR, emotions are distinguishable through both respiratory and cardiac activity (for anger, fear, sadness, and happiness, see Rainville, et al.^68^). Changes in heart rate (HR) correlate with the perception of emotional stimuli: the averaged beats per minute (BPM) or mean HR decelerates with the viewing of negative items compared with either positive or neutral ones^69–72^. The emotional intensity or arousal, paired with an elevated mean HR, predicts better memory performance^73,74^. Furthermore, the EDA amplitude is enhanced for emotional pictures compared with neutral ones^75^ and changes in the EDA famously correlate with the emotional intensity^76,77^.

Similarly, the implication of the self in events, especially through the self-reference effect^78–80^, has a positive influence on memory: self-related stimuli tend to be better remembered than other-related ones (see for instance, for a recent study, Moses-Payne, et al.^9^), and that effect is still present even when the self is slowly eroding (e.g. in Alzheimer’s disease^81,82^). In VR, a few studies have studied the self in relationship with memory (for a recent review, see Bréchet^83^), hinting at a link between the bodily self and autobiographical memory^84^. Combined with an enhanced sense of presence in VR, the virtual enactment effect, stemming from sensorimotor and cognitive interaction in a VE, is defined as the effect on memory retrieval of one or more dimensions among “motor commands, proprioceptive information, vestibular information, decision-making, and allocation of attentional resources”^85^. Virtual enactment tends to have a positive effect on memory, as well as the sense of presence in embodied conditions, from a full-body avatar seen in a mirror to no avatar, which can be improved by sensorimotor involvement^86^. Nevertheless, manipulating some aspects of immersion does not always influence memory^87^. Like emotions, the self can also be measured through bodily signals^88^, including the heart rate and skin conductance. Changes with EDA correlate with the self-versus other perspective, as does the brain-generated neural response at each heartbeat^89^. More precisely, the neural responses to heartbeats in the precuneus and the medial prefrontal cortex co-vary respectively with the ‘I’ and ‘Me’ dimensions of the self^90^, i.e., the experiencing self as the moment-to-moment awareness of being, and the conceptual self which encompasses one’s identity, personal traits, and self-knowledge^5^.

Interestingly, there is strong evidence going towards shared ground between emotional and self-related processes. Gutchess and Kensinger^91^ put a new model forward in which encoding processes for self-related and emotional information overlap through shared prioritised processing during encoding. Both emotions and self-reference, according to the authors, benefit from common processes such as attention (either prioritised or sustained), elaboration and refreshing of information. These processes play a key role in the encoding of emotional, self-related information, by capturing and sustaining attention and, subsequently, elaborating on it and refreshing it. Common neural markers supporting these shared mechanisms have been put forward: the medial prefrontal cortex and the late positive potential. Besides, both emotions and self-related processes lead to an enhanced recall, reciprocally to more vividness and more positive self-related information recalled. It is noteworthy that this co-influence of emotions and self-reference on long-term memory is influenced by other factors, such as age with the positivity bias, toward building and maintaining a positive self, increasing with aging (see for instance Kalenzaga, et al.^81^); and even though both older and younger adults tend to have a positive view of their self or self-concept, negative items or events are more reliably and vividly recalled in younger adults^63^. Likewise, a change in emotional state may also alter this interaction between emotions and the self in memory (e.g., depression^92,93^).

Building on previous research, the present study aimed at determining which factors present during naturalistic encoding influence the retention of personally experienced events in long-term memory (i.e., EAM). Using immersive VR, participants experienced a walk through a virtual city from a first-person perspective, where they encountered 30 events (i.e., dynamic scenes) with various emotional valences (positive, neutral, negative), which they could either interact with or only observe (actor, observer). The encoding was incidental and physiological measures were recorded during immersion (HR mean, respiration mean, EDA activity). The participants did three unexpected free recalls of the events: one immediately after encoding, one a week after encoding, and one a month after encoding. Each event recall was scored for the presence of accurate *what*-*where*-*when*elements and subjective details, which was used as a measure of EAM performance. The free recall at a month was followed by a recognition test. Furthermore, at the end of each recall session, the participants rated the events on subjective scales to evaluate diverse relevant dimensions of memory^30^ such as the emotional content, their point of view actor/observer for the event, the self-relevance, the anticipated details, the mental images, the frequency of real-life encounters.

Our main purpose was to determine what dimensions, among the subjective scales of the events at the immediate recall, would best explain how events are remembered immediately, one week and one month after encoding. First, we evaluated the evolution through time of the recall scores and subjective assessments. We expected a decline in the subjective scales and recalls over time, in line with previous literature^30,94–96^. Second, we investigated the role of subjective assessments of the events following the immediate recall in predictive models of recall through time. We hypothesised that the subjectively assessed emotional content and perspective, as well as the self-relevance of the events, would appear prominently in the models featuring the subjective assessments; however, we also expected additional variables to contribute to later recalls. We aimed at further explaining the one-month recall using the physiological data, that we expected to be linked with the subjective scales, especially those related to emotion and the self.

## Methods

### Participants

Healthy participants were recruited through the academic network platform of the RISC-UAR 3332 CNRS (Relay of information on the sciences of cognition), the professional social networks of members of the laboratory, and among medical school students of Université Paris Cité. Exclusion criteria were signs of depression (score > 15 on the Beck Depression Inventory^97^), high travel sickness (score > 7 on the Simulator Sickness Questionnaire^98^), and anxiety (score > 65 on the State-Trait Anxiety Inventory-Form Y^99^). Further exclusion criteria included a psychiatric, neurological, or cardiac condition, head trauma, drug addiction, recent neurosurgery, pacemaker, the need for a respiratory help device, paralysis and amputation, pregnancy, frequent dizziness, vertigo, or nausea, being a free diver and being susceptible to develop severe COVID-19 symptoms. All participants had normal or corrected-to-normal vision and audition and were French speakers; they took no alcohol, drugs, or medications prior to their visits to the laboratory. All participants gave written informed consent and were compensated for their participation (10€ per hour). The study was approved by the Research Ethics Committee of the University Paris Cité (CER U-Paris Cité) (N° IRB: 00012021-60), in accordance with the Declaration of Helsinki. It was also authorized by the Data Protection Officer (DPO) and registered in the processing activities of Université Paris Cité (FR-UPC-20220401-MC²Lab_DLD_P), following the RGPD guidelines.

A total of 30 participants was planned to be included in the study, based on similar previous studies examining long-term memory retention via VR (e.g., Foudil, et al.^25^, Penaud, et al^84^., or Girardeau, et al^100^.). A power analysis for a Repeated Measures ANOVA (within factors; simple main effect of time) indicated that the minimum sample size to yield a statistical power of at least 0.8 with an alpha of 0.05 and a medium effect size (f = 0.25) is 28. Among the 34 participants who came for the encoding session, three experienced and reported cybersickness and, therefore, could not complete the first session (8.82% total cybersickness); one participant did not complete the second and third sessions and was excluded from the analyses.

In total, 30 participants completed the whole experiment. They were aged 18 to 40 years old (M = 26.27, SD 7.08; Female ratio: 66.67%) and mostly were students (63.33%). Four of them were left-handed (13.33%).

## Materials

### VR material

The incidental encoding took place in a virtual environment built with Unity 3D 2019.2.5f1 in the lab. The participants were equipped with a VR head-mounted device (HTC Vive Pro). The environment was a multisensory town with auditory inputs during pre-determined events (conversations, noises…), but without the background noise of a town so that participants would not get distracted. The town was similar to Paris, featuring similar architecture and some famous monuments (Eiffel Tower, train station such as the Gare de Lyon, Montparnasse Tower…), enhanced by a series of dynamic scenes featuring objects and avatars (see below, encoding phase, for further details). While the participants walked in the town, no cars drove in the streets both to ensure the safety of the participants and to allow them to focus completely on the task at hand. In the town, the participants had to follow a pre-established white luminous line on the pavement. They could “walk” using the touchpad of one of the two HTC controllers (left controller) they held. To change direction in the VE they had to turn on themselves in real life, while remaining on the spot. They could interact with some objects in the environment with the other controller: these objects were lightly highlighted in yellow. For a few events, they could use a smartphone to take a short recording or call someone. In that case, the smartphone highlighted in yellow appeared in the air next to them so that they could use it. In the VE, the participants could see their hands, represented with black gloves. When they pressed a button on a given controller, the chosen hand contracted; when they used a touchpad, the thumb of the chosen hand followed their movement.

## Physiological data instrumentation and processing

A series of physiological data were recorded only during the encoding task. They were wirelessly recorded using the Biopac MP160 acquisition system, including two surface electrodes on the non-dominant hand (index and middle finger) for the EDA recording, two surface electrodes on the right clavicula and under the ribcage on the left side for the ECG recording, and a respiratory belt. Signals were recorded during the virtual walk on the Biopac Acqknowledge software. They were strictly synchronised with the events as they happened for the participants. All the signal processing was done using the Acqknowledge software. The EDA, ECG and respiration recordings were first resampled to 1000 samples per second (linear interpolation method). The EDA and respiration signals were then smoothed and filtered (low-pass FIR filter for EDA, frequency cutoff: 1 Hz; bandpass FIR filter for respiration: between 0.05 Hz and 1 Hz). The respiration rate was then computed, and the means for each event (from its start to its end: 20 s duration) was calculated for the analyses. Likewise, the HR was computed based on the qrs peaks, and its mean for each event was calculated. The EDA processing was a little different: with the phasic EDA, the skin conductance response (SCR) events were computed from a period beginning one second after the onset of each event and ending ten seconds after the onset. This period of time was chosen as the SCR is not instantaneous, and neither were the events. A ten-second period provided enough time for an EDA response to happen if there were to be one.

## Additional material

The following scales were used to check VR immersion and its impact:

- Emotional state: Befindlichkeits-Skala, BfS, Bobon, et al.^101^ – in which the participants chose between two options or neither for 28 pairs to describe their current state, such as ‘relaxed’ or ‘stressed’, ‘in a very good shape’ or ‘miserable’ (as translated from French; scores can reach 0 to 56);
- Cybersickness: Simulator Sickness Questionnaire, Kennedy, et al.^98^ – in which participants rated 16 cybersickness-related items as experienced during VR on Likert scales (0 to 3), such as general discomfort, nausea, vertigo or tiredness;
- Embodiment : Piryankova, et al.^102^ – in which participants rated 14 embodiment-related items as experienced during VR on Likert scales (0 to 5), such as their perception of their virtual hands in the VE or body ownership;
- Presence : Makowski, et al.^103^ – in which participants rated 14 presence-related items as experienced during VR on Likert scales (0 to 5), such as the interest in their surroundings in the VE or the feeling of being there.

### Procedure

**Fig. 1:**
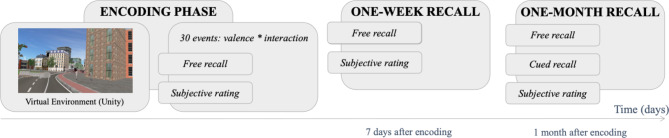
Framework of the experimental procedure. During the incidental encoding phase, the participants either witnessed or interacted with 30 specific events of various emotional valences that they subsequently tried to recall (free recall) and rated on several subjective scales (all events rated at the end of the encoding phase and at the end of the one-month session; only freely recalled events rated at the end of the one-week recall). They did a second free recall a week later and the last one a month after encoding.

The experiment comprised three main phases (Fig. 1): the encoding phase with the first free recall, a second free recall and a third free recall. The second free recall happened a week after the encoding session (M = 7.77 days, SD = 1.17), and the last one a month after encoding (M = 29.53 days, SD = 3.18). The first session (encoding and first free recall) lasted around two and a half hours, and the two recall sessions at a week and a month lasted around one hour. At the end of the first and second sessions (end of encoding session and end of one-week session), the participants were asked to come back for some ‘baseline measurements’ for the experiment and were not given the indication that they would be further interviewed about their memory of the VR walks. Hence, in total, the memory of 30 events was tested on 30 subjects at 3 different time points.

## Encoding protocol & measures

*Pre-VR evaluation*: The encoding phase was preceded for each participant by a series of standard scales and cognitive tests (not included in the present study but used to check the equivalence of participant groups in future studies) and a short training session in the VE. After these scales and tests, and having listened to the VR instructions, the participants did a VR training session to ensure they understood how to navigate in the environment and use highlighted objects and the phone. To that aim, they experienced mock events: one observation, one interaction with a phone and one interaction with highlighted objects, all while following the pre-established white luminous line. The training session happened in the same VE as the main experiment, though in different streets.

### VR session

**Fig. 2:**
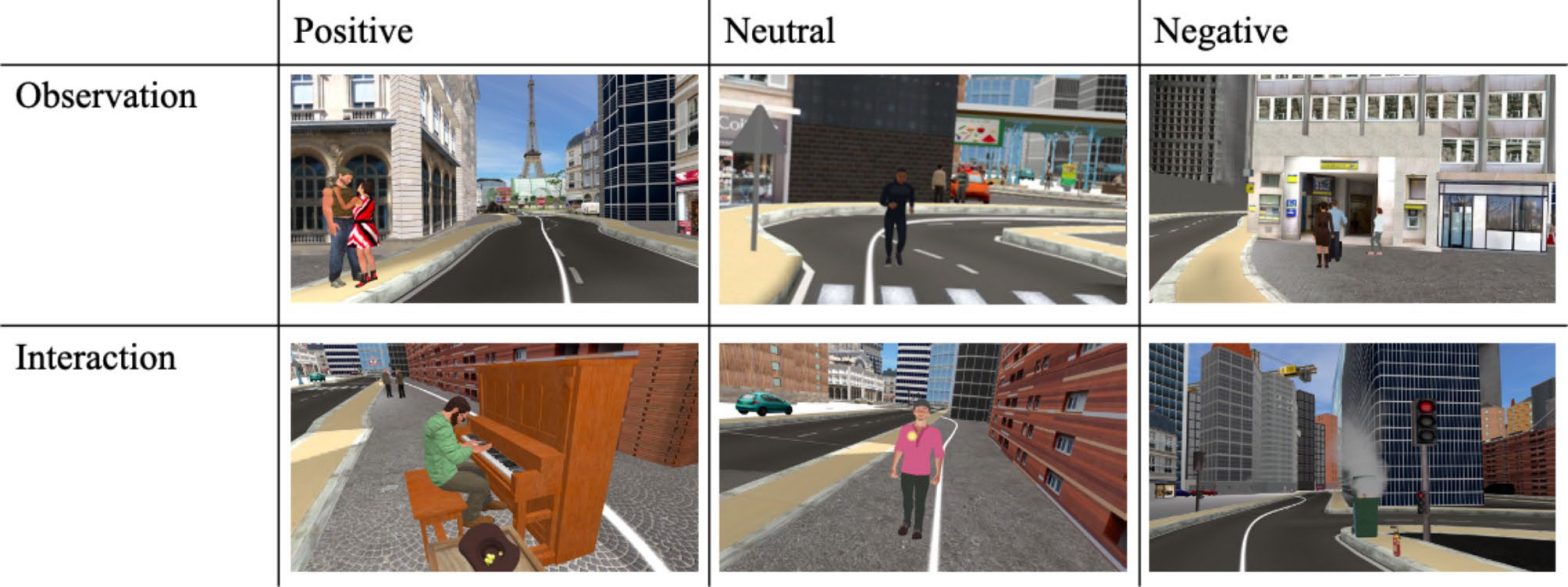
Examples of events in the virtual environment with their main different characteristics. Positive observation: “A couple kisses on the street.” Neutral observation: “A jogger runs past”. Negative observation: “In a line in front of the post office, a woman loses patience. She decided to cut the line while complaining about the situation”. Positive interaction: “A man is playing the piano beautifully; the participant puts a few coins in his hat”. Neutral interaction: “A man asks what time it is; the participant shows him the locked screen of their phone to answer”. Negative interaction: “The participant walks near a trash can that catches fire. They try to put it out with an extinguisher, but it does not work (though the fire is still contained)”.

The participants were asked to explore the city by following the path, and mentally assess whether they generally liked this environment and whether they would enjoy doing their daily activities or living there. Along the path, the participants encountered 30 realistic events in total (see Supplementary Table S1for their description). In order to create relevant personal experiences in VR, the events were unique and situated in a specific spatio-temporal context. They had different valences: 12 positive, 12 negative and 6 neutral; and could either be interacted with (highlighted object) or simply observed: 15 interactions and 15 observations. The specificity and emotional value of the events had previously been assessed in the laboratory^84^. All events had both visual detailed components and an audio track with appropriate sound (voices, noises related to the events…). They each lasted 20 s, during which the participants were stopped in front of the event. The participants could only go back on the white-line path once the 20 s and the event were over. Examples of events are provided in Fig. 2. The events were uniformly split into three sets. Each of these 10-event sets constituted one track in which the events were in a pre-established order, avoiding two consecutive events with the same characteristics. The three tracks were shown in a counterbalanced order and the participants spent 10 to 15 min in each; in the VE, they ended with a ‘STOP’ sign. Participants were encouraged to report any sign of cybersickness; in this case, the experience would come to an end to prevent any unwelcome effects.

### Post-VR evaluation

After the VR session, the levels of embodiment^102^, presence^103^, and cybersickness^98^during VR were assessed. Even if they reported higher-than-normal levels of cybersickness, they were not excluded from further analyses if they had been able to complete the experiment. Their emotional state (Befindlichkeits-Skala, BfS^101^) was assessed before VR and immediately after the training session and the three VR tracks to control for any negative emotional impact due to VR immersion. Then, the participants did the first immediate free recall (approximately 10 min after the end of the VR session). The immersion in the VE, training session comprised, lasted one hour.

## Recall protocol

**Fig. 3:**
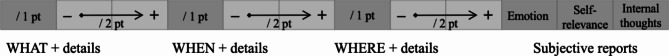
Quotation of the memories. The episodic score (EM) is the sum of What, Where, When and their details (maximum of 9), the episodic autobiographical score (EAM) is the sum of the EM score and the subjective reports, which are the reported emotion, self-relevance, and internal thoughts (maximum of 12).

*Free recalls*: This experimental protocol involved three surprise memory tests, the validity of which had been checked with previous VR studies from our laboratory (e.g., Penaud, et al.^84^). The recall phase was the same at each of the three points: immediately after encoding (after the post-VR evaluation), one week and one month after encoding. Each time, the memory of the events was assessed through a free recall. The participants were instructed to provide verbally, over a maximum 10-minute period, as many events as they could from their virtual walk, with as many details as they could: what happened, when and where it happened, what they thought and felt emotionally, whether the event related to them in any way. No feedback was provided about any free recall. A standard EAM scoring method was adapted to that task^3,104^, based on previous studies in ecological^30^ and VR^84^ paradigms (see Fig. 3). For *what*, *where* and *when* respectively, one point was attributed for the correct mention of an element from the considered category, or zero in the case of incorrect or no information (i.e., the binary *what*/*where*/*when* scores). The binding scores *what-where*, *what-when*, and *what-where-when*, was equal to one if each of the binary scores of the considered binding score was equal to one; otherwise, it was equal to zero (maximum of one for each binding score). With the possibility of earning up to two additional points for providing further details, a maximum of three points was thus attributed for each of the following categories: *what*, *where*, *when*. The subjective reports category also had a maximum of three points: one point for the emotion, one for the internal thoughts, one for the relevance to one’s self. The addition of the *what*, *where* and *when* categories gave the EM score (maximum of 9 points per event recalled); the EAM score was the addition of the EM score and the subjective reports category (maximum of 12 points per event recalled) (see Supplementary Materials for a detailed example of the scoring of an event).

### Recognition

The last recall session also comprised a recognition task: participants were shown complete pictures of the events, as shown in the VE and therefore in context, and had to tell whether they had encountered them during their navigation and provide a Remember / Know / Guess (RKG) judgement for each ‘Yes’ answer^105^. Among the pictures shown, 25% of the total number shown (40) were lures with the same visual properties and with similar types of events as the ones in our VE, set in the same VE: for instance, people discussing, a person playing the guitar on the street, a man standing on the way, a dog… The participants did not have a time limit to recognise the pictures in this old/new RKG recognition (one static image from an event spanning several seconds).

### Subjective assessments

After each recall session, the participants rated all the events on 12 subjective scales (Table 1). These Likert scales, ranging from 0 (‘no, not at all’) to 5 (‘yes, very much’), were inspired by previous EAM studies covering different retention delays (e.g., Piolino, et al.^28^; Viard, et al.^29^). More precisely, the participants were asked to ‘rate the different events that they encountered during the virtual walk’ (translated from French). For the one-week recall, they only rated the events they had evoked during the free recall to avoid any re-encoding of forgotten items; for the two other recalls, all the events were rated. For the one-month recall, the subjective assessments were done after the recognition. For all the subjective assessments, the events were shown as Graphics Interchange Format or GIFs (short, repeating videos) on a computer, with a grey, uniform background, and did not comprise the audio part of the VR experiment. At the end of the last two recall sessions, participants also filled a questionnaire about factors of influence of consolidation (tiredness, sickness, traumatic events, internal and external repetition of the VR session, impact of the pandemic; see Table S2), and a second questionnaire about the experiment general understanding, clarity, and enjoyment.

**Table 1:** List and description of the subjective assessments of the events. The variables were subjectively assessed by the subjects on scales from 0 to 5 for each item at the end of the encoding session and at the end of each recall session.

Variable	Description
Emotional intensity	Intensity: emotional intensity
Emotional valence	Valence: emotional valence, whether the event is pleasant or unpleasant
Self-relevance	Personal importance of the event, whether the event is personally significant
Remembering	Knowing or remembering, whether one knows the event happened or actively recollects it
Perspective	Observer or actor perspective, whether one observes the images in the memory or whether one sees them as they lived the event
Self-concept	Characterisation: event characteristic of oneself, how the event reflects personality, objectives, desires
Thinking	Internal: anticipated frequency of mental repetition over the following month
Conversation	External: anticipated frequency of external repetition over the following month, with friends and family for instance
Anticipated details	Prediction of details still present in a month, precision of the event in memory in a month of time given the number of details available
Reliving	To what extent the event is relived with details
Mental images	Number of mental images prompted by this event
Frequency of real-life encounter	Whether the event can be often encountered in real life

### Data analyses

The data was analysed using R^106^ (version 4.4). The missing data on subjective scales (related to the absence of EAM recalls), concerning only the one-week subjective assessment as the participants did not rate every event (410 missing assessments out of 900), was replaced using a Principal Component Analysis (PCA) (missMDA package^107^). Rather than imputing missing data using the means, thus creating considerable distortions in the dataset, we used an imputation with this method. It evaluated the links between individuals and between variables, and imputed missing data through a regularised, iterative PCA algorithm (see for instance, Loisel and Takane^108^ for a comparison with other methods). Of note, we replaced our missing data at a week with a method that allowed us to preserve the trends of the dataset. Nonetheless, this point should be kept in mind when considering the results involving the one-week subjective assessments, as a little under half of the data was initially missing. Separately, for the physiological measures, recorded during encoding, such a replacement was not possible; any missing data from these variables at encoding was thus replaced by the mean for the corresponding event. The Psych package^109^ was used for the descriptions of the dataset.

First, **control checks**were carried out. The Grubbs (outliers package^110^) test was used on the one-month EAM free recall, averaged on the participants, to control the presence of any outlier for both the highest and the lowest scores. To conform with local ethical demands and control any potential negative influence of the manipulation of emotional valence on participants, we reviewed whether the emotional state of the participants was altered after VR compared to before VR (repeated measures ANOVA); we also reviewed the answers the participants provided to satisfaction questions. The correlations between consolidation questions and the corresponding free recall scores were checked, as well as the correlations between the recall scores and cybersickness, presence, and embodiment. The correlations with the subjective assessments at encoding was evaluated for the physiological data. The lmer function from the lmerTest package^111^ was used for this last check (Bonferroni family-wise correction, initial ⍺ of 0.05).

Second, we investigated the **evolution of subjective assessments and memory scores through time**(repeated measures ANOVAs for time, based on linear mixed effects models with random intercepts for the participants, on the dataset averaged on the participants considered as the items; Bonferroni family-wise alpha correction). Tukey pairwise comparisons were done using emmeans^112^.

Then, we performed **predictive models on each free recall EAM score and on recognition**, as our main dependent variables, **explained by all the subjective assessments**as rated after the first immediate recall during the encoding session. Linear mixed models were fitted by residual maximum likelihood using the lmer function from the lmerTest package^111^. Participants and events were modelled with a random intercept (models run on the complete, not averaged dataset), and t-tests for significance were computed using Satterthwaite’s method. Conditional and marginal R^2^ were computed using the multilevelTools package^113^. The conditional R^2^provides an estimation of the variability explained by the model; the marginal R^2^provides the same estimation, but only for the fixed effects. The recognition alone was investigated using logistic models (generalized linear mixed models fit by maximum likelihood, Laplace Approximation) for old trials with hit rates coded as 1 and misses coded as 0; for the recognition, an estimation of the R^2^ was made using an equivalent mixed model.

Finally, **predictive models on each free recall EAM score and on recognition explained by each physiological measure** (models on each memory score separately for HR, respiration rate, EDA latency, EDA amplitude and presence or absence of EDA response) were investigated using linear mixed effects correlations (participants and events with random intercepts, same as above) to assess whether the memory performance could be explained, or predicted, by these objective measures recorded live during encoding. The physiological data was thus included separately in the analyses as its main objective was to assess, separately from all other subjective variables, whether it was correlated with the main variable of interest (the one-month free recall EAM score). This allowed us to have on the one hand, models with ‘subjective’ variables, i.e., the subjectively assessed scales; and on the other, models with ‘objective’ variables, i.e., our physiological data.

Unless otherwise specified, the recall score considered as the dependent variable was the total EAM score.

## Results

### Control check

*Outliers*: Based on the data, at the 5% significance level, our results did not reject the hypothesis that both the lowest and the highest values are not outliers (highest value: G = 1.99, U = 0.859, *p* = 0.610; lowest value: G = 2.70, U = 0.739, *p* = 0.0591). In other words, as we could not quantitatively conclude that either value was an outlier, no participant was excluded from the analyses.

#### Emotional state of the participants

**Fig. 4:**
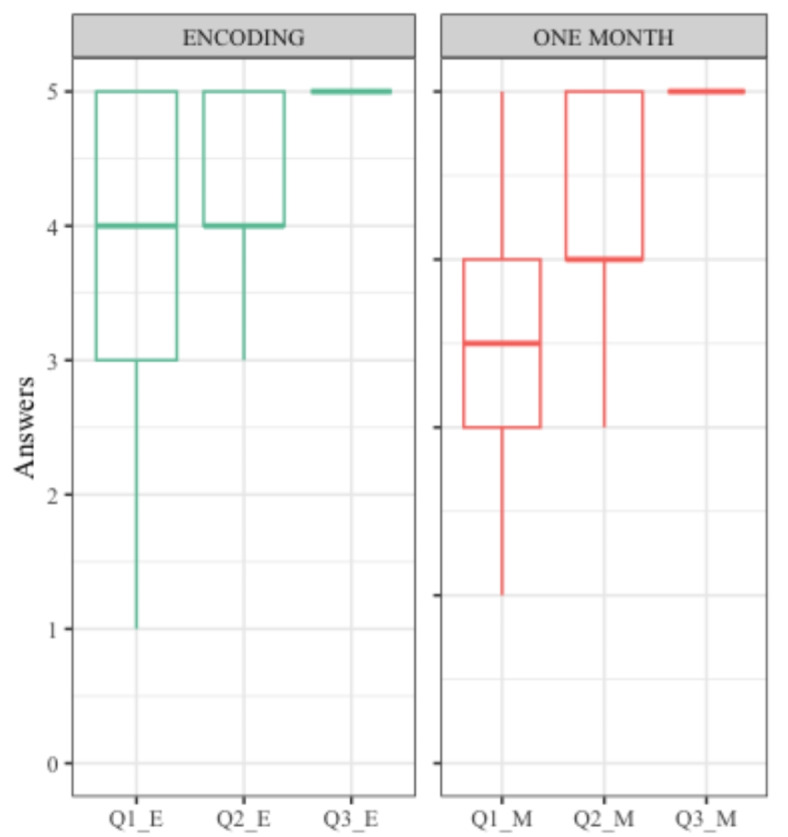
Satisfaction questions for the immediate and one-month sessions, rated on a Likert scale from 0 (not at all) to 5 (a lot / perfectly). Boxplot: starting at the minimum, with the first quartile, median, third quartile, to the maximum. Questions translated from French. At the end of the first session (encoding phase): Q1_E: How much did you like the walk in the city? Was the environment pleasant? (M = 3.80, SD = 1.19; Min: 1, Max = 5). Q2_E: Did you enjoy using the VR setup? (M = 3.90, SD = 1.16; Min: 0, Max = 5). Q3_E: Were the instructions clear? (M = 4.93, SD = 0.25; Min: 4, Max = 5). At the end of the last session (end of the experiment): Q1_M: Today, did you enjoy recalling what you saw in the city? (M = 3.47, SD = 0.86; Min: 2, Max = 5). Q2_M: Did you enjoy your participation in this experiment? (M = 4.27, SD = 0.69; Min: 3, Max = 5). Q3_M: Were the instructions of this last session clear? (M = 4.80, SD = 0.76; Min: 1, Max = 5).

The emotional state of the participants did not significantly differ before and after the VR session (no significant difference for the BfS, F(1,29) = 0.03, *p* = 0.864). They reported good to very good levels of satisfaction and enjoyment regarding the first and the last session (Fig. 4).

#### Consolidation questions

The one-month EAM free recall score did not correlate with the consolidation questions (see list of questions in Table S2); the one-week EAM free recall score only correlated with Question 5 (‘Did you experience a particularly striking event over the period of time since you last came to the lab?’) in a limited way (r(28) = 0.395, *p* = 0.0308 before alpha correction). The EM scores did not correlate with the consolidation questions.

#### Link with cybersickness, presence, embodiment

The free recall scores (EM and EAM) did not correlate with the cybersickness, presence, or embodiment scores at each time point (*p* > 0.05 before alpha correction, see Table S3).

#### Link between subjective assessments at encoding and physiological measures

Before alpha correction, only the EDA latency significantly correlated (negatively) with subjective assessments at encoding, namely the self-relevance, self-concept, thinking, and anticipated details (see Table S4 for the estimates and p-values of each correlation).

### Effect of time

Participants freely recalled, as a mean, around 54% of the events, i.e., 16.1 (SD = 4.06, min = 6, max = 22), 16.5 (SD = 4.85, min = 4, max = 24), and 16.1 (SD = 4.53, min = 4, max = 23) of the 30 original events freely recalled at the immediate, one-week and one-month tests. They correctly recognised 88% (mean number of recognised events for the hit rate: 26.47, SD = 3.48, min = 15, max = 30) of the events at the one-month delay. Out of 900 memory tests (30 participants x 30 events), 339 were recalled at every test (immediate, one-week, one-month free recall and recognition). Table 2 provides the EAM means for each recall (means for all events and all participants and means for only the recalled events), which indicated higher means when taking into account only the recalled events at each time point rather than all events.

**Table 2:** Assessments of memories from encoding to the last delayed retrieval: results of the repeated measures ANOVAs for time (linear mixed models with random intercepts for subjects, dataset averaged on participants). The range (min: max) and number of observations for the EAM scores are also provided. Alpha correction: adjusted ⍺ level of 0.004, Bonferroni family-wise correction of alpha inflation. The significant effects after alpha correction are in bold. Tukey post-hoc tests (pairwise comparisons): T1: significant difference only between immediate and one-week recall.

Variable	Immediate recall	One-week recall	One-month recall	Statistic	Raw *p*-value	Effect size	Post-hoc test
	*Mean (SD)*	*Mean (SD)*	*Mean (SD)*	*F(2*,*58)*	*p-value*	*Partial η²*	
**EAM /12 (all events)**	2.39 (2.60) – 0:10–900	2.46 (2.59) – 0:9–900	2.40 (2.63) – 0:9–900	0.247	0.782	/	/
**EAM /12 (recalled events)**	4.46 (1.84) – 1:10–483	4.47 (1.78) – 1:9–495	4.47 (1.9) – 1:9–483	0.0181	0.982	/	/
**EM /9**	2.04 (2.28)	2.15 (2.26)	2.06 (2.26)	0.621	0.541	/	/
**What total /3**	1.09 (1.10)	1.10 (1.07)	1.08 (1.08)	0.0422	0.959	/	/
**Where total /3**	0.58 (0.92)	0.71 (0.97)	0.62 (0.93)	3.87	**0.0263**	0.12	T1
**When total /3**	0.37 (0.76)	0.34 (0.74)	0.36 (0.74)	0.193	0.824	/	/
**Emotion**,** Thoughts**,** Self /3**	0.35 (0.64)	0.31 (0.61)	0.34 (0.64)	0.892	0.416	/	/
**What (binary) /1**	0.54 (0.50)	0.55 (0.50)	0.54 (0.50)	0.273	0.762	/	/
**What details /2**	0.55 (0.67)	0.55 (0.64)	0.55 (0.64)	0.0458	0.955	/	/
**Where (binary) /1**	0.29 (0.46)	0.36 (0.48)	0.31 (0.46)	3.60	**0.0335**	0.11	T1
**Where details /2**	0.29 (0.48)	0.35 (0.50)	0.31 (0.48)	3.97	**0.0242**	0.12	T1
**When (binary) /1**	0.21 (0.41)	0.19 (0.39)	0.20 (0.40)	0.382	0.685	/	/
**When details /2**	0.16 (0.39)	0.15 (0.38)	0.15 (0.38)	0.0718	0.931	/	/
**Emotion /1**	0.15 (0.36)	0.15 (0.36)	0.15 (0.36)	0.066	0.936	/	/
**Self /1**	0.09 (0.29)	0.10 (0.32)	0.11 (0.32)	0.915	0.406	/	/
**Thoughts /1**	0.10 (0.30)	0.06 (0.24)	0.07 (0.26)	3.67	**0.0315**	0.11	T1
**What-Where /1**	0.294 (0.456)	0.356 (0.479)	0.314 (0.465)	3.60	**0.0336**	0.11	T1
**What-When /1**	0.208 (0.406)	0.19 (0.393)	0.202 (0.402)	0.382	0.685	/	/
**What-Where-When /1**	0.146 (0.353)	0.132 (0.339)	0.137 (0.344)	0.269	0.765	/	/

#### Time & subjective assessments

Overall, the subjective scores, assessed by the participants at the end of each session and described in Table 1, decreased over time (*p* < 0.001 for most subjective scales). The results of this analysis are presented in Table 3. Regarding the emotional intensity, the score initially increased and then decreased (*p* = 6.30e-10). Concerning the self-relevance, perspective, self-concept, and frequency of real-life encounter, scores decreased from the immediate and one-week levels to the one-month level; scores concerning remembering, thinking, conversation, mental images and reliving decreased from the immediate to the one-week and one-month levels. There was no significant effect of time only on the anticipated details, emotional valence and frequency of real-life encounter after alpha correction (see Table 2).

**Table 3:** Subjective assessments of memories from encoding to the last delayed retrieval: results of the repeated measures ANOVAs for time (linear mixed models with random intercepts for subjects, dataset averaged on participants). Each variable is rated on Likert scale (0 to 5). Alpha correction: adjusted ⍺ level of 0.004, Bonferroni family-wise correction of alpha inflation. The significant effects after alpha correction are in bold. Tukey post-hoc tests (pairwise comparisons): T2: each pairwise comparison is significant; T3: non-significant difference between immediate recall and one-week recall, significant differences otherwise; T4: non-significant difference between one-week and one-month recall, significant differences otherwise; T5: significant difference only between one-week and one-month recall.

Variable	Immediate recall	One-week recall	One-month recall	Statistic	Raw *p*-value	Effect size	Post-hoc test
	*Mean (SD)*	*Mean (SD)*	*Mean (SD)*	*F(2*,*58)*	*p-value*	*Partial η²*	
**Emotional intensity**	2.07 (1.52)	2.36 (1.28)	1.82 (1.59)	31.21	**6.30e-10**	0.52	T2
**Emotional valence**	2.50 (1.60)	2.52 (1.40)	2.39 (1.60)	3.91	0.0255	0.12	T5
**Self-relevance**	1.86 (1.64)	1.87 (1.40)	1.54 (1.60)	15.10	**5.25e-06**	0.34	T3
**Remembering**	2.78 (1.56)	2.45 (1.18)	2.20 (1.69)	15.26	**4.74e-06**	0.34	T4
**Perspective**	3.21 (1.54)	3.16 (1.15)	2.78 (1.75)	7.90	**0.000924**	0.21	T3
**Self-concept**	1.61 (1.62)	1.68 (1.44)	1.33 (1.55)	13.65	**1.39e-05**	0.32	T3
**Thinking**	1.40 (1.28)	1.25 (1.17)	0.98 (1.25)	8.46	**0.000597**	0.36	T3
**Conversation**	1.20 (1.35)	0.69 (1.05)	0.71 (1.15)	16.64	**1.94e-06**	0.36	T4
**Anticipated details**	1.87 (1.38)	1.81 (1.17)	1.76 (1.50)	0.513	0.602	/	/
**Reliving**	2.96 (1.58)	2.50 (1.27)	2.46 (1.72)	15.56	**3.89e-06**	0.35	T4
**Mental images**	2.50 (1.41)	2.16 (1.19)	2.01 (1.53)	20.07	**2.37e-07**	0.41	T4
**Frequency of real-life encounter**	2.51 (1.61)	2.47 (1.34)	2.27 (1.58)	5.09	0.00922	0.15	T3

#### Time & free recall scores and subscores

**Fig. 5:**
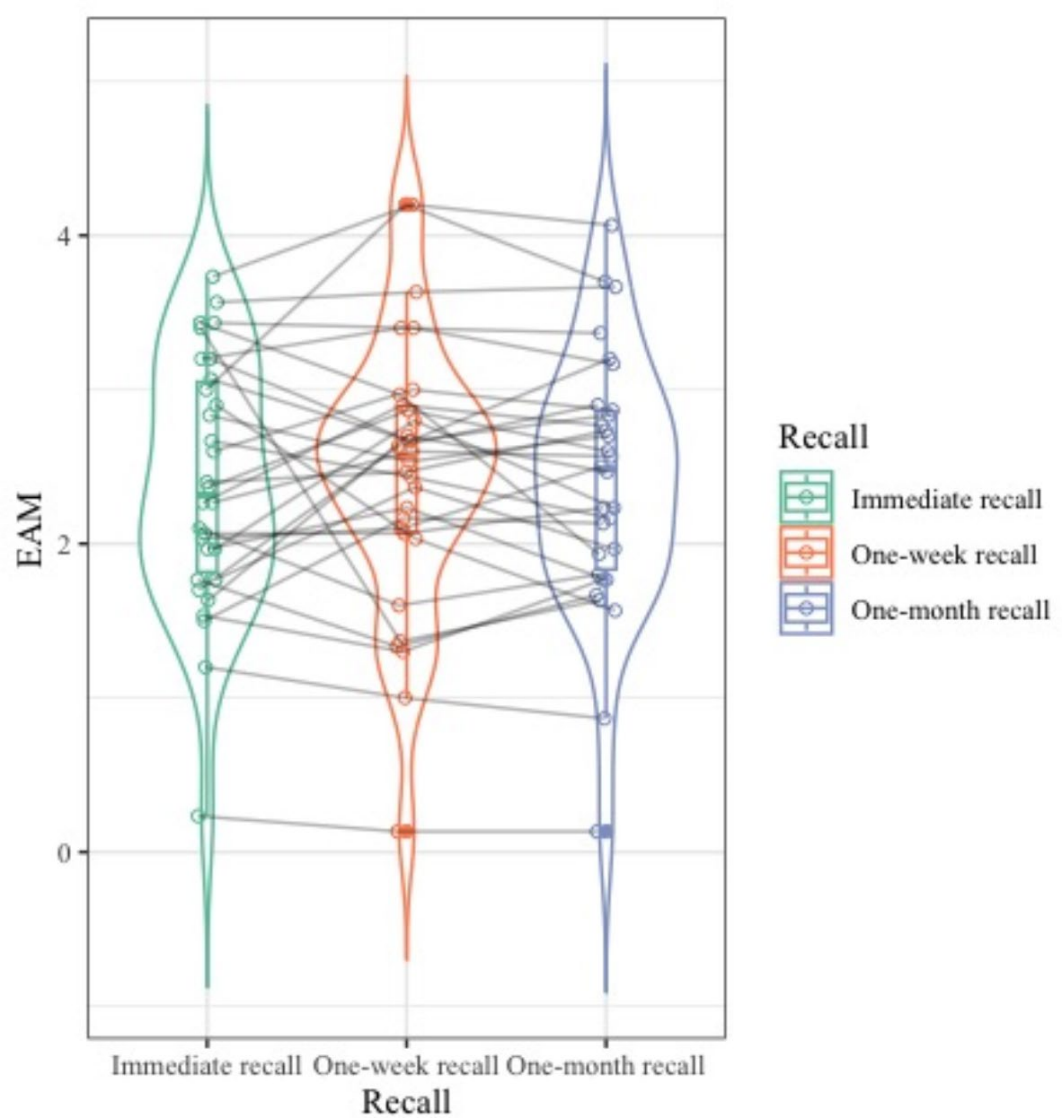
Evolution of the free recall memory score over time (EAM score). For ease of reading in the graph, the means of the scores for each participant are represented. Violin plot: coupled with a box plot, the width corresponds to the frequency of the considered data point.

On the other hand, there was no clear evolution through time for the free recalls, either EAM or EM, and binding scores (Fig. 5 presents the evolution through time of the mean EAM score of each event; results can be found in Table 2). Notably, only the total *where* score with its subscores (*where* correct answer and *where* details), as well as the *what*-*where* binding score, significantly increased (before alpha correction) at the one-week recall with no difference between the immediate and one-month recall levels. The score for the internal thoughts followed the same trend.

#### One-month recognition

The participants correctly recognised 88% of the presented pictures during the one-month recognition test, the hit rate being associated with 80% of Remember, 13% of Know and 7% of Guess responses. 12% of the lures lead to false alarms (see Table 4).

**Table 4:** Recognition test at a month: proportions of recognition hit (hit rate) on the events, false alarm on the lures (representing 25% of the presented pictures) and proportions, among the recognised items, of the ‘Remember’, ‘Know’ and ‘Guess’ answers. Sensitivity: d’ = 2.51.

Recognition hit			False alarm		
88%			12%		
**R**	**K**	**G**	**R**	**K**	**G**
80%	13%	7%	36%	14%	50%

### Prediction analyses of memory

#### Based on subjective assessments

The predictive models based on all the subjective assessments of the 30 events, as rated after the immediate free recall at the end of the first session, showed that some variables were significant predictors. Table 5 presents the coefficients and p-values of the variables that significantly contributed to each model.

##### Immediate recall prediction

As explained by the subjective assessments at the end of the first session, the immediate free recall EAM score was explained by remembering, thinking, and frequency of real-life encounter. Only the frequency of real-life encounter was a negative predictor. The fixed and random effects explained 33.2% of the variability of the model.

##### One-week prediction

In the model, the one-week free recall EAM score was explained by the following factors as assessed after the immediate free recall: emotional valence, remembering, mental images, and frequency of real-life encounter. Both the frequency of real-life encounter and the emotional valence were negative predictors. The fixed and random effects explained 25.5% of the variability of the model.

##### One-month prediction

Taking the same variable as the predictors, the one-month free recall EAM score was explained by the same factors, with the additional factors of thinking and conversation. The frequency of real-life encounter, emotional valence, and conversation were negative predictors. The fixed and random effects explained 22.4% of the variability of the model.

##### Recognition prediction

For this model, only the reliving variable was a significant predictor. The coefficients of the model are reported in Table S5. The fixed and random effects explained 23.0% of the variability of the model.

**Table 5:** Results of the models. Models were run with the total recall score of immediate recall, one-week recall or one-month recall as the dependent variable, and the first set of subjective scorings (after the immediate recall) as predictors or independent variables. The p-values of the significant predictors are in bold.

	Immediate recall			One-week recall			One-month recall		
**Marginal R** ^**2**^	0.218			0.122			0.117		
**Conditional R** ^**2**^	0.332			0.255			0.224		
	β	95% CI	*p*	β	95% CI	*p*	β	95% CI	*p*
**Emotional intensity**	-0.0149	[-0.165; 0.140]	0.846	0.0983	[-0.0578; 0.258]	0.222	0.0939	[-0.0657; 0.257]	0.255
**Emotional valence**	0.0187	[-0.100; 0.137]	0.760	-0.157	[-0.285; -0.0284]	**0.0175**	-0.151	[-0.284; -0.0188]	**0.0250**
**Self-relevance**	-0.0302	[-0.164; 0.106]	0.661	0.0192	[-0.120; 0.159]	0.789	0.0366	[-0.106; 0.180]	0.619
**Remembering**	0.403	[0.225; 0.583]	**1.21e-05**	0.194	[0.00964; 0.379]	**0.0407**	0.306	[0.114; 0.496]	**0.00181**
**Perspective**	0.150	[0.00112; 0.299]	0.0503	0.0837	[-0.0699; 0.239]	0.289	0.0416	[-0.116; 0.203]	0.603
**Self-concept**	0.100	[-0.0310; 0.234]	0.138	0.107	[-0.0297; 0.245]	0.128	0.0794	[-0.0609; 0.222]	0.273
**Thinking**	0.346	[0.162; 0.526]	**2.09e-04**	0.115	[-0.0756; 0.302]	0.233	0.210	[0.0139; 0.402]	**0.0340**
**Conversation**	-0.0824	[-0.243; 0.0762]	0.313	-0.0765	[-0.246; 0.0883]	0.364	-0.170	[-0.344; -0.00145]	**0.0491**
**Anticipated details**	0.128	[-0.0391; 0.290]	0.130	-0.00590	[-0.178; 0.163]	0.946	-0.0238	[-0.201; 0.149]	0.790
**Reliving**	-0.0380	[-0.220; 0.137]	0.662	0.00803	[-0.177; 0.191]	0.932	-0.0206	[-0.211; 0.167]	0.831
**Mental images**	0.130	[-0.0361; 0.298]	0.129	0.244	[0.0717; 0.418]	**0.00609**	0.183	[0.00692; 0.364]	**0.0445**
**Frequency of real-life encounter**	-0.182	[-0.297; -0.0669]	**2.31e-03**	-0.189	[-0.313; -0.0658]	**0.00304**	-0.129	[-0.258; -0.00257]	**0.0470**

### Based on physiological measures

*Immediate recall prediction*: As a trend, the immediate free recall score was explained only by the HR mean (trend: *p* = 0.0819) and the EDA amplitude (trend: *p* = 0.0936).

#### One-week prediction

As a trend, the one-week free recall score was explained only by the HR mean (trend: *p* = 0.0833) and the EDA response (trend: *p* = 0.0567).

#### One-month prediction

The one-month free recall score was significantly explained by the presence of an EDA response (β = 0.355, CI 95% [0.00468; 0.706], *p*= 0.0471; marginal R^2^of 0.00453, conditional R^2^ of 0.147), showing a better memory performance when there is an EDA response. However, it was not mediated by the other physiological variables (non-significant fixed effects for EDA latency, amplitude, HR mean, and respiration mean).

#### Recognition prediction

The one-month recognition was predicted by the respiration mean (β = -0.00437, CI 95% [-0.00634; 0.00446], *p*= 6.7e-06; estimated marginal R^2^of 0.000151, estimated conditional R^2^ of 0.201) and EDA amplitude (β = -0.584, CI 95% [-1.13; -0.0358], *p*= 0.0368; estimated marginal R^2^of 0.00518, estimated conditional R^2^ of 0.274), showing a better memory performance with a decreased respiration mean, and with a lesser EDA amplitude.

## Discussion

The encoding of EAM in real-life settings may involve various concurrent factors of influence, mainly related to emotions and the self. This innovative study aimed at exploring the formation of EAM in an ecological context, controlled by the experimenter, and to determine which encoding factors and subsequent subjective assessments of the events would predict the events’ memory outcomes (EAM scores), which take into account both the presence of *what*, *where*, *when* elements, and subjective details. We investigated the long-term recall of EAMs in young, healthy adults, who had incidentally encoded specific events of varying emotional valences in VR, either by interacting with them or observing them, while simultaneous physiological data was recorded. Three unexpected free recall memory tests and subjective assessments happened: immediately after encoding, one week, and one month after encoding. The main findings showed that subjective assessments generally declined over time, whereas no such effect was found for the EAM scores, except for spatial elements and internal thoughts. The one-month EAM free recall score was explained by several variables as assessed after the immediate free recall, including the emotional valence, prospective aspects (external and internal repetition), the frequency of real-life encounter, and mental images. The recording of physiological data during encoding provided a significant clue as to whether the events would be remembered a month later. This study had two main objectives that will be discussed below: (a) investigating the evolution through time in memory of ecologically encoded events and (b) determining what factors could predict recall through time, based on subjective assessments of each event after encoding and physiological recordings during encoding. The results of this study are discussed in relation to the insight gained into the formation of the episodic autobiographical memory trace.

### Evolution through time

The first goal of this study was to assess the evolution through time of the subjective assessments and free recall scores of controlled naturalistic events. To be as close as possible to real-life situations, we used an incidental encoding paradigm mimicking the formation of the memory trace in real-life situations, exposing participants to events of varying emotional valences and interactions (i.e., observer or actor) in an immersive virtual urban environment. The involvement of the self and emotion in the encoding process are two well-established elements that determine the formation of a long-lasting trace in autobiographical memory^114,115^(see for instance the case of flashbulb or vivid memories: Conway, et al.^32^, Talarico and Rubin^116^).

Both the mean total EM and the EAM scores, as well as the mean EAM score of only the recalled events, were not found to change over time, with around 54% of events recalled at each time point: nevertheless, the *where* component showed a variation with a slim increase at the one-week evaluation. At the end of the last session, the participants reported not having thought or talked much about the experienced VR session (see Table S2; this did not correlate with the prospective internal and external rehearsals: internal rehearsal: r(28) = 0.199, *p* = 0.293; external rehearsal: r(28) = 0.163, *p*= 0.391). The absence of a proven change in free recall performance over time may hint at memory permanence or even consolidation (see for instance a previous VR study interpreting no decrease of event memory over time, after sleep, as consolidation: Abichou, et al.^117^), as this could indicate no forgetting of past personal experiences when they are immediately recalled and refreshed by subjective assessments (though this has to be proven yet for the present study; however, see the testing effect and Roediger and Abel^118^). Interestingly, the improvement in the spatial component at one week highlights its central role in consolidation^119,120^. For instance, during consolidation, sleep has been found to improve spatial memory^121^, and EM^117^in young adults, notably through hippocampal replay^122^. Exploring EM in VR thus allows to target, among others, this specific component of memory. Here, an increase of the *what-where*binding score was found at the one-week recall, in line with studies about consolidation of spatial associative memory^123^. These results about the *where*scores seem to be in line with previous findings about the role of spatial replay after learning (both in rodents^124^and humans^125^), and more specifically about the richness of EAM. The spatial component is one powerful clue of a detailed, recollective retrieval in EAM relying notably on the hippocampus, cuneus and precuneus; the consolidation and strengthening of this aspect thus contributes to long-term EAM retrieval^53,119,126^.

By contrast, the subjectively assessed scores (subjective scales) for the events globally decreased over time, while the *internal thoughts*subscore of EAM increased at a week. This points to the reminiscence of the knowledge of one self’s thoughts during encoding and to the phenomenological recollective experience, by oneself, of an event that was experienced by the self in the past. This passage of phenomenological information from the encoding experience to knowledge about the past links the former experiencing ‘I’ with the experienced self ‘me’ at the centre of EAM recall^115^. In the present study using immersive VR, in an ecological setting resembling daily-life environments, participants experienced specific and relevant events in a first-person perspective which allowed us to assess fundamental aspects of EAMs. Furthermore, the decline of the subjective assessments of the events is consistent with previous literature, and more particularly with the recency effect of the retention curve of EAM described by Rubin^96^(see also Piolino, et al.^95^). A slight persistence of the factors related to the Self and the emotion was found, in line with previous findings^30^. Indeed, in the present study, the emotional intensity, as well as the self-relevance, self-concept and perspective globally decreased mostly from the one-week level to the one-month level, reflecting the importance of emotion^60,127,128^ and self-relevance^129,130^ in encoding and the first stages of consolidation. The ratings of these factors, highly linked with autobiographical memory, have previously been reported as constant through time^131^.

### Prediction of long-lasting memory from encoding

Our second goal was to investigate potential predictors at encoding of the EAM at different time points. In line with Gutchess and Kensinger^91^, who described a model with shared processes during encoding for both self-reference and emotion, with common electrophysiological potentials and areas of activation in the brain, we expected that subjective measures of the self and emotion associated with the experienced events (see also flashbulb and vivid memories^116^), as well as physiological measures, would be predictors of long-lasting EAM.

Common variables were found from the subjective assessments at encoding, which predicted EAM through time, that is, at the immediate, one-week and one-month recalls: the sense of remembering and the frequency of real-life encounter of the event. The importance of the sense of remembering from encoding to long-term retention highlights the conscious recollection of personal experiences, beyond the merely known or familiar, as a key factor in the formation of EAM and further retrieval. Delayed recognition was explained by the strength of the sense of reliving from encoding, which can be seen as a deeper form of remembering where the memory is re-experienced in a rich, vivid and immersive way. The senses of remembering and reliving are linked to the concepts of EAM^3^, mental time travel^2^and self-memory system^132^. More particularly, mental time travel allows to mentally relive the event as if it were happening again, with its rich sensory and emotional details. Otherwise, the frequency of real-life encounter of the events substantially predicted whether the events would subsequently be remembered: the less an event was reported as being frequently encountered, the better it would later be remembered. In this study, saliency of the events was thus an important factor during encoding to predict the memory status at both a week and a month, in line with a consistent body of literature (see for instance Rubin and Kozin^31^; arousal-biased competition theory, Mather and Sutherland^133^; and the SLIMM model for incongruent items, van Kesteren, et al.^134^).

Immediately after encoding, EAM was additionally predicted by the anticipated internal thinking about the experienced events. At one-week, additional processes appeared with the emotional valence and mental images, but not thinking. At a month, memory rehearsal, both internal and external, was again added to the prediction. The influence of emotional valence on EM and EAM is well-known^61,135^(see Introduction). It is noteworthy that emotional valence plays a role only later in the consolidation process, from the one-week recall onward. While emotion influenced EAM recall, this result specified a timeframe within which emotionally charged content begins to affect the consolidation process of EAM itself. This delayed impact of emotional valence as a predictor suggests that emotional processing continues after the encoding phase, likely during consolidation, and thus helps to explain why emotionally salient memories tend to endure longer^135^. This seems to confirm the importance of such a later recall. The present study also showed that subjectively assessed negative events are better remembered. In young adults, negative emotion has previously been reported to enhance memory, as well as the ability to recall internal details such as thoughts of low-intensity events^136^.

Mental images also played an important role, reflecting their general importance in cognition^137^and the importance of mental scene construction in autobiographical memory^138,139^, of the quality of the mental image of the event (remembering) at the heart of EAM. In particular, mental imagery of scenes is linked with subjective elements of EAM^3^and shares fundamental neural activations with EM, making mental imagery a credible predictor of memory^140^. Besides, the internal rehearsal (mentally revisiting the memory, self-reflection about the event) had a mixed role, as it positively predicted both the immediate and one-month EAM free recall scores, but the rehearsal with peers (discussing or retelling through conversation) was a negative predictor of the one-month free recall. Both internal and external repetitions are well-known processes of memory consolidation. This dual impact of rehearsal highlights how self-reflection and social sharing are crucial for retaining vivid autobiographical memories over time^141^. However, external rehearsal is also considered through the semanticisation of EAM, since uniqueness and specific details are reduced in favour of generic memories^23,142^. Moreover, this simultaneous influence of external and internal reactivation could hint at a higher role of more internal processes in consolidation, such as mind-wandering which could promote episodic future thinking and EAM re-consolidation through spontaneous or voluntary mental imagery (see for instance on this question Girardeau, et al.^100^); and at the involvement of the narrative self (linked with the construction and coherent maintenance of one’s identity through time, by integrating personal experiences into life stories^132^) with a potential selection of the to-be-remembered events with a self-narrative and self-centred rather than other-centred focus^143^. The involvement of these prospective, consolidation-related variables in the immediate and one-month but not the one-week prediction could hint at the involvement of supplementary, more long-term processes, in the farther rather than the intermediary recall, such as an influence of episodic future thinking during encoding on subsequent long-term recall.

It is however surprising that except for a sense of remembering, internal rehearsal or reliving, no other self-related variables in the subjective assessments (e.g., self-relevance, self-concept) influenced memory performance in this study. In a previous experiment, we found a strong role of self-related subjective assessments in predicting EM for real-life events^30^, such as the sense of remembering, reliving, self-concept, perspective. A possible explanation could be that the events, as salient and/or emotional they could be, were not personally relevant enough. Moreover, the participants did not actively choose their path in the VE, preventing any positive influence of pro-action^38^, and neither did they choose the events they would experiment, removing any self-choice effect^144^. Furthermore, here, the participants mostly rated their point of view in their memories of the events as a first-person perspective that they adopted for their recall of the events. Research suggests that first-person memories tend to be more vivid, emotionally intense, and rated higher on the subjective feeling of reliving. This heightened level of detail and emotional intensity in first-person memories may lead to stronger recollection and more durable memory over time^145^; with a different task or different VR modalities, the role of the self in predicting EAM might thus differ.

These behavioural results were in line with the physiological measures. These measures made it possible to predict what would become, in the participants’ memories, of the events experienced: the presence of an electrodermal response could forecast, even while explaining less variability than the subjective assessments (i.e., 14.7% versus 22.4% of variance explained), how rich the EAM trace of the event would be one month later, and both the respiration mean and the EDA amplitude satisfyingly predicted the recognition assessment (around 27% of variability explained). Among the physiological measures, only the EDA latency was negatively correlated with some subjective assessments, mostly related to the self. In other words, the more an event was rated as having a high self-relevance and self-concept, the faster the EDA response would be. Likewise, the response would be faster for some prospective elements: anticipated details and internal thinking. The EDA latency thus directly reflects elements of EAM: prospection, and the relationship with the self^88,89^. On the contrary to previous literature, we did not find a direct link between emotion and HR or respiration means^68,70^, neither with EDA amplitude^75^. Neither HR nor respiration correlated with the self. However, future studies should more frequently combine physiological measures with ecological tasks in VR to better understand the exact extent of these effects, which could be linked with objective factors of the events.

### Temporal dynamics of EAM

All things considered, the present study highlights the temporal dynamics of mechanisms underlying EAM. A common, core set of characteristics could be identified in the predictive variables from the encoding phase, particularly in terms of the sense of remembering and the frequency of real-life encounter of the event. These indicators, reflecting both the strength of recollection and novelty of the event, were strong predictors of EAM retention, spanning from an immediate to a long-lasting recall up to a month. These findings emphasise the importance of phenomenological, subjective experiences in memory retention, reinforcing the notion that the quality of initial encoding – in terms of sensory richness and personal significance – is critical for memory longevity. It is suggested that EAM is highly dependent on how richly detailed and self-engaging the original event was, which is key to its retention over time. Other processes appeared later in time. While these core predictive factors remain constant over time, they alone were not enough to maintain a rich long-term trace. Other processes emerging from the encoding phase became predictive depending on the timing of the recall. For example, emotional valence, with a negativity bias observed in young adults, became a predictor of EAM after one week, as well as mental imagery. Additionally, anticipated external and internal rehearsals from the encoding phase came to be predictors of the one-month recall, with the internal rehearsal also contributing to the immediate recall.

The novelty of these results regarding EAM formation lie in several key aspects related to the temporal dynamics of predictive variables. Namely, different predictive variables become relevant at different points in time. This dynamic, time-sensitive shift between memory predictors challenges the traditional view that encoding factors uniformly influence memory retention across all time points. The fact that predictive variables shift and evolve highlights the multiphasic nature of memory formation, indicating that distinct mechanisms operate not only during encoding but also during consolidation and retrieval. This temporal emergence of various predictors and the absence of a significant decline in EAM scores over one month support both the multiple-trace theory of memory consolidation^119,146^ and the trace-transformation theory^147–150^. These theories propose that memory consolidation is not a static process but rather a dynamic and ongoing process, ensuring its long-term stability. The integration of novel elements during reactivation helps prevent forgetting and adds to the complexity of EAM formation. The models suggest that memory consolidation involves supplementary mechanism that stem from the original encoding, which intervene during both the consolidation phase and each instance of retrieval or reactivation. These processes preserve a rich memory trace over extended retention periods. Without these ongoing processes, deeply tied to real-life-like phenomenological, subjective, and personal experiences, memory recall would degrade over time, primarily due to deficiencies in the encoding stage. Thus, our results reinforce the idea that memory is an adaptative, evolving process with each recall or reactivation involving supplementary mechanisms from the original encoding, rather than a static phenomenon where factors influencing retention are determined solely at encoding and exercise their influence evenly through time^91^. Mechanisms of influence come and go, and new elements are likely introduced over time to counter natural forgetting, shaping the formation of long-lasting EAM^151^. This study offers strong support for contemporary theories like multiple-trace theory and expanding our understanding of how EAM forms and persists over time.

### Strengths, limits and perspectives

In summary, our results provide substantial new insight into how memories are formed in a complex, naturalistic, real-life-like environment in which specific events of varying emotional valence are either observed or interacted with. Using an ecological, naturalistic setting while still maintaining control from the experimenter is a near-perfect balance for the exploration of the formation of EAM. To our knowledge, no other study using VR investigated EAM directly from encoding, combining objective measures of interest in the VE and subjective reports about one’s own experience in the VE, and assessing memory performance in the long-term. One strength of this study regarding the prediction of EAM formation is the evidence that different predictive variables become relevant at different points in time. The emotional intensity and valence, the frequency of real-life encounter, prospective elements (thinking and conversation), and mental images have a critical role in predicting memory at a one-month recall. The addition of supplementary processes in the prediction of memory through time has seldom been studied before (for a delayed role of recognition at a retention delay of 24 to 72 h, see Sharot and Yonelinas^135^). To our knowledge, this longitudinal study provides first elements of a comprehensive understanding of the prediction of EAM through time based on a wide array of subjective variables pertaining to the personal cognitive experience of the encoded events.

Nevertheless, this experiment had some limits that should be addressed in further studies. Here, the participants were mainly young university students. Thus, the generalisation of the findings should be considered with caution. Moreover, a larger cohort of participants would allow further exploration of the interactions between time and the characteristics of the events experienced in the VE. Notably, with 30 events in total, we avoided ceiling effects and despite the complexity of the immersive task, we limited the levels of cybernetic discomfort that would have prevented the experiment’s completion^152^. In contrast, the results might indicate a floor effect, as the participants demonstrated a moderate level of performance in the free recall tasks, suggesting a low richness of memories with this VR paradigm. In the present study, with an incidental encoding in a complex VR setting for both the VE and the hardware, the cognitive load associated with the task might have been very high and might have prevented optimal performance, leading to a potential floor effect. This limitation was moderated by an 88% hit rate recognition and 54% of events free recalled (see Mair, et al.^18^: the number of details for participants who did not review the material before recall was not as high as the other participants in a review condition). Furthermore, when only the recalled events were considered, the performance was observed to be largely superior, indicating the maintenance of rich EAM over time in our VR paradigm compared to what would be expected by classic laboratory material^153–155^. In the present study, the high percentage of recalled events in a month hints at real-life-like processes at work in memory consolidation for this task rather than more laboratory-based ones^156,157^. Moreover, even though they may not constitute a floor effect, the low scores can also be explained by the challenge of the free recall task (free recall of unrelated events without cues), and the scoring which takes into account the richness of the trace and not only its presence. An intentional rather than incidental encoding could lead to stronger performances^158^, but would be less ecological, examining learning capacities rather than naturalistic encoding. The explained variance of the models declined over time: 33.2% for the immediate recall, 25.5% for the one-week recall and 22.4% for the one-month recall. While still satisfying at one-month, this hints at the involvement of additional processes in explaining long-term EAM that were not investigated or manipulated in this study, including factors related to the self.

To complete this exploration, virtual but highly self-related events could be considered: for instance, memories with flashbulb-like characteristics^32,159,160^, that is, important, highly emotional, generally collective and often surprising events of which the context of encoding is subsequently highly recalled; or encoded events chosen or built by the participant (self-choice effect, see for instance Baldwin, et al.^144^). Moreover, modulating the level of presence, embodiment, and choice, for instance by varying how immersive the hardware is, and by allowing the participants to choose the route they follow, would be interesting to observe whether, in this paradigm, these factors would have some other direct influence on EAM. Furthermore, in the present experiment, three surprise recalls took place. Even though no information was given regarding subsequent recalls (see Methods), some of the participants might have anticipated the recalls, but this issue was not controlled during the experimental procedure. Applying this paradigm to another recall group without the two intermediary tests, thus limiting potential processes of voluntary re-consolidation, could help determine the extent of this effect, as the participants would then have no potential information that there would be subsequent memory tests^161^; moreover, it could allow to determine whether the explaining factors of the one-month memory performance would be the same without rehearsal through recall and re-consolidation. Future studies should also consider longer delays to control how much would then be recalled^162^, and different age groups to explore the feasibility of this paradigm with older participants, as age is well-known to have an effect on EM and EAM^104,163^. Also, different well-known factors of influence of consolidation, e.g., the quality of sleep, were not investigated here but should be more thoroughly examined in future longitudinal studies, or more regularly probed during the long-term retention delay. In a clinical perspective, the mere presence of an electrodermal response informs about what will become of the event in memory, and this could prove useful in contexts where the collection of subjective assessments is not possible. Training in VR using physiological measures (as done for instance by Bögge, et al.^164^), and highlighting important points of interest, corresponding to the predictors in the present study, to focus on for patients, could lead to interesting results in enhancing memory encoding and preventing memory loss.

In conclusion, by using a realistic VR setting and ecological specific events with the simultaneous manipulation of both the emotion and the interaction during encoding, this experimental paradigm provides new avenues for EAM exploration from encoding, and could have potential applications in clinical settings.

## Electronic supplementary material

Below is the link to the electronic supplementary material.

Supplementary Material 1

## Data Availability

The raw data supporting the findings of this study, as well as the code used and written for the analyses, will be made available by the authors (corresponding authors: DL, PP) upon reasonable request.
