# Supplementary material for: Decoding episodic autobiographical memory in naturalistic virtual reality
Source: Sci Rep. 2024 Oct 27;14:25639. doi: 10.1038/s41598-024-76944-3 (PMC11514229; doi:10.1038/s41598-024-76944-3)
Supplement: Supplementary file 1 — Supplementary Material 1 [file 41598_2024_76944_MOESM1_ESM.pdf]

Supplementary materials

Description	Valence	Interaction
A young man gives his place to an older man to sit on a bench.	+	–
A couple kisses on the street.	+	–
A group of children chats and laughs (one of them imitates a gym teacher from school).	+	–
Next to a verdant tree, birds fly away, and a few leaves fall.	+	–
An ATM machine lets out bank notes, a woman retrieves them happily.	+	–
A man announces they just won the lottery and rejoices.	+	–
A man is playing the piano beautifully; the participant puts a few coins in his hat.	+	+
The participant tries a fortune wheel; they win, and a woman applauds.	+	+
A young female singer lets the participant choose a song (either "Don't stop me now" by Queen or "I'm still standing" by Elton John) and sings it.	+	+
A show in the street with a woman dancing and a man playing the violin; the participant takes a picture with a smartphone.	+	+
A child congratulates the participant on their tee-shirt; the participant and the child high-five.	+	+
The participant throws back a basketball to two male players who thank the participant.	+	+
A male jogger runs past.	N	–
A man does jumping jacks and stretches his arms.	N	–
A woman steps in her car.	N	–
A man asks what time it is; the participant shows him the locked screen of their phone to answer.	N	+
In front of a newsstand, the participant reads a newspaper and then put it back.	N	+
A button is pushed by the participant to make the light turn green and cross the street.	N	+
An older woman in a wheelchair crossed the street; her wheelchair creaks.	–	–
In a line in front of the post office, a woman loses patience. She decided to cut the line while complaining about the situation.	–	–
In front of a smoking bakery, the baker laments the loss of their business.	–	–
A rat comes from under a garbage bin and runs on the pavement.	–	–
A homeless man, who staggers a little, asks for a coin.	–	–
A female manager of a store fires two employees and illegally asks for their letter of resignation.	–	–
The participant walks near a trash can that catches fire. They try to put it out with an extinguisher, but it does not work (though the fire is still contained).	–	+
A man falls on the street, two women assist him. The participant is able to call for rescue on their smartphone.	–	+
In front of a stand on the street with ancient object, the participants break a vase when they interact with it. The vendor is upset and sad.	–	+
A dog barks threateningly until a tennis ball is thrown to distract it.	–	+
The participant swats flies away from garbage in the middle of the street.	–	+
A man steals personal belonging from a woman who calls for help; the participant is able to call for rescue on their smartphone.	–	+

Table S1: List of the thirty events with their associated valence (+: positive; N: neutral; –: negative) and interaction (+: active interaction; –: passive interaction, observation).

**At the end of the second session (end of the one-week recall)**	
Q1: How frequently have you been thinking about the VR session that you did when you last came to the lab? (0: never; 5: very frequently)	M = 2.43, SD = 1.22; Min: 0, Max = 4
Q2: How frequently have you talked about the VR session that you did when you last came to the lab? E.g., to friends, colleagues, your family… (0: never; 5: very frequently)	M = 2.07, SD = 1.39; Min: 0, Max = 5
Q3: Generally speaking, how would you rate your tiredness over the period of time since you last came to the lab? (0: very tired; 5: feeling great)	M = 3.03, SD = 1.40; Min: 0, Max = 5
Q4: Have you been sick since you last came to the lab? (0: not at all; 5: a lot, regularly)	M = 0.37, SD = 0.93; Min: 0, Max = 3
Q5: Did you experience a particularly striking event over the period of time since you last came to the lab? E.g., traumatic event, big life step, huge news from someone close… (0: nothing; 5: exceptionally striking event)	M = 1.57, SD = 1.68; Min: 0, Max = 5
**At the end of the last session (end of experiment)**	
Q1: How frequently have you been thinking about the VR session that you did when you last came to the lab?	M = 2.23, SD = 1.14; Min: 0, Max = 4
Q2: How frequently have you talked about the VR session that you did when you last came to the lab?	M = 1.97, SD = 1.19; Min: 0, Max = 4
Q3: Generally speaking, how would you rate your tiredness over the period of time since you last came to the lab?	M = 2.60, SD = 1.28; Min: 0, Max = 5
Q4: Have you been sick since you last came to the lab?	M = 0.60, SD = 1.07; Min: 0, Max = 4
Q5: Did you experience a particularly striking event over the period of time since you last came to the lab?	M = 1.83, SD = 1.56; Min: 0, Max = 5
Q6: Did the public health crisis related to the pandemic affect you? (0: not at all; 5: a lot)	M = 1.80, SD = 1.42; Min: 0, Max = 5
Q7: Do you think the health crisis may have influenced how you did the experiment? (0: not at all; 5: a lot)	M = 0.30, SD = 0.79; Min: 0, Max = 4

Table S2: Consolidation questions, rated on a Likert scale from 0 to 5. Questions translated from French.

	**EM scores**						**EAM scores**					
	**Immediate**		**One-week**		**One-month**		**Immediate**		**One-week**		**One-month**	
	β	p	β	p	β	p	β	p	β	p	β	p
**Embodiment**	-0.00575	0.628	-0.00163	0.888	-0.00873	0.43	-0.000672	0.955	0.00150	0.909	-0.00517	0.674
**Presence**	3.118e-04	0.983	0.0111	0.429	0.00426	0.753	0.00758	0.603	0.0158	0.320	0.0107	0.476
**Cybersick-ness**	-0.0154	0.574	0.0317	0.232	0.0226	0.378	0.00789	0.776	0.0510	0.0851	0.0458	0.1

Table S3: Correlation matrix for cybersickness, presence and embodiment. With linear mixed-effect models: fixed effects for the variables of interest, random intercepts for the subjects and events. Raw p-values reported. Initial α of 0.05: here, Bonferroni correction provides a corrected α of 0.003.

Variable	HR mean	Respiration mean	EDA latency	EDA amplitude	EDA response
**Emotional intensity**	β = 0.269, t = 0.809, p = 0.419	β = -0.00170, t = -0.019, p = 0.985	β = -0.0793, t = -1.87, p = 0.0625	β = 0.00703, t = 0.707, p = 0.48	β = 0.0154, t = 1.26, p = 0.208
**Emotional valence**	β = 0.00840, t = 0.025, p = 0.98	β = 0.0818, t = 0.982, p = 0.327	β = 4.146e-04, t = 0.009, p = 0.993	β = 0.0120, t = 1.14, p = 0.255	β = 0.00741, t = 0.597, p = 0.551
**Self-relevance**	β = 0.262, t = 0.937, p = 0.349	β = -0.0230, t = -0.30, p = 0.764	**β = -0.0979, t = -2.75, p = 0.00611**	β = 0.00352, t = 0.429, p = 0.668	β = 0.0108, t = 1.04, p = 0.297
**Remembering**	β = 0.435, t = 1.37, p = 0.17	β = 0.0667, t = 0.765, p = 0.444	β = -0.00250, t = -0.066, p = 0.947	β = 0.00450, t = 0.490, p = 0.624	β = 0.00598, t = 0.519, p = 0.604
**Perspective**	β = 0.431, t = 1.23, p = 0.221	β = 0.105, t = 1.09, p = 0.277	β = 0.0575, t = 1.48, p = 0.139	β = 0.00259, t = 0.257, p = 0.797	β = -0.0169, t = -1.35, p = 0.178
**Self-concept**	β = 0.207, t = 0.736, p = 0.462	β = 0.00385, t = 0.05, p = 0.96	**β = -0.0904, t = -2.48, p = 0.0132**	β = 0.00818, t = 0.986, p = 0.325	β = 0.00525, t = 0.504, p = 0.614
**Thinking**	β = 0.463, t = 1.26, p = 0.207	β = -0.112, t = -1.11, p = 0.267	**β = -0.0907, t = -2.01, p = 0.0449**	β = 0.0186, t = 1.74, p = 0.0819	β = 0.00768, t = 0.572, p = 0.568
**Conversation**	β = 0.0101, t = 0.028, p = 0.978	β = -0.132, t = -1.32, p = 0.188	β = -0.0408, t = -0.924, p = 0.356	β = 0.0111, t = 1.04, p = 0.298	β = 0.0135, t = 1.02, p = 0.31
**Anticipated details**	β = 0.201, t = 0.597, p = 0.551	β = 0.0595, t = -0.603, p = 0.547	**β = -0.101, t = -2.41, p = 0.0164**	β = 0.0145, t = 1.48, p = 0.139	β = 0.0113, t = 0.917, p = 0.359
**Reliving**	β = 0.461, t = 1.41, p = 0.158	β = 0.0595, t = 0.666, p = 0.506	β = 0.00474, t = 0.126, p = 0.9	β = 0.00389, t = 0.415, p = 0.678	β = 0.00216, t = 0.184, p = 0.854
**Mental images**	β = 0.486, t = 1.45, p = 0.148	β = 0.0592, t = 0.643, p = 0.52	β = 0.0193, t = 0.463, p = 0.644	β = -0.00178, t = -0.182, p = 0.856	β = 0.00704, t = 0.572, p = 0.567
**Frequency of real-life encounter**	β = 0.373, t = 1.12, p = 0.262	β = 0.0477, t = 0.564, p = 0.573	β = -0.0126, t = -0.279, p = 0.78	β = -0.00191, t = -0.186, p = 0.853	β = -0.0128, t = -1.05, p = 0.293

Table S4: Control checks. Correlations between the physiological data and the subjective assessments at encoding (linear mixed effect models, with Satterthwaite's method, participants and events with random intercepts). Alpha correction: adjusted α level of 0.0008, Bonferroni family-wise correction of alpha inflation.

	**Recognition**		
**Marginal R^2**	0.0760		
**Conditional R^2**	0.230		
	β	95% CI	*p*
**Emotional intensity**	0.166	[-0.106; 0.439]	0.231
**Emotional valence**	-0.139	[-0.365; 0.0859]	0.225
**Self-relevance**	-0.0941	[-0.344; 0.155]	0.460
**Remembering**	-0.113	[-0.444; 0.218]	0.502
**Perspective**	0.0796	[-0.169; 0.328]	0.530
**Self-concept**	-0.0779	[-0.325; 0.169]	0.536
**Thinking**	0.196	[-0.151; 0.544]	0.268
**Conversation**	-0.163	[-0.441; 0.114]	0.248
**Anticipated details**	0.168	[-0.153; 0.489]	0.305
**Reliving**	0.319	[0.0162; 0.622]	**0.0390**
**Mental images**	0.242	[-0.0611; 0.545]	0.118
**Frequency of real-life encounter**	-0.0142	[-0.210; 0.181]	0.887

Table S5: Results of the model for the recognition test. Models were run on the 'old' trials with hit rates coded as 1 and misses rated as 0, and the first set of subjective scorings (after the immediate recall) were used as predictors or independent variables. The p-values of the significant predictors are in bold.

Example – scoring of an event

Transcripts translated from the French recordings: Negative event, no interaction.
Description: In front of a smoking bakery, the baker laments the loss of their business.

At the immediate free recall: “So, one of the events I remember is the garbage bin that was on fire. It was on my right side. The extinguisher was right before it, but I did not see it at first. So, yes, I put out the fire. And then, I was, it evoked some sensations… First, I was stressed when I saw the fire. Then, when I could see [the extinguisher] was not successful, I felt unsafe or even like in real life.”

Scoring: Total = 5 for EM score (out of 9); 6 for EAM (out of 12)

What: 1; What details: 2
Where: 1; Where details: 1
When: 0; When details: 0
Emotion: 1; Self-relevance: 0; Internal thoughts: 0

At the one-week free recall: “Then, I remember the garbage bin that was on fire. I needed to take… It was in front of me, slightly on the right, rather. I had to take a fire extinguisher and put out the fire, but it didn’t work. So as an emotion, I felt fear.”

Scoring: Total = 4 for EM score (out of 9); 5 for EAM (out of 12)
What: 1; What details: 1
Where: 1; Where details: 1
When: 0; When details: 0
Emotion: 1; Self-relevance: 0; Internal thoughts: 0

At the one-month free recall: “Then, I remember some garbage bins that were on fire, and I had to put out the fire. I had fire extinguishers on my right-hand side, I was surprised and stressed.”

Scoring: Total = 4 for EM score (out of 9); 5 for EAM (out of 12)
What: 1; What details: 1
Where: 1; Where details: 1
When: 0; When details: 0
Emotion: 1; Self-relevance: 0; Internal thoughts: 0

Information about the scoring procedure:
The scoring procedure was agreed upon by DL, IM, and PP, and based on previous validated scoring procedures (e.g., Lenormand et al., 2022, Penaud et al., 2022, 2023). Each recalled event, for each participant, was scored separately, with no knowledge of the corresponding subjective assessments, that is, no knowledge of the participants’ evaluations of the variables of interest. Here, the procedure only takes into account objective elements (is a given piece of information, which are all verifiable in the VE and identical for each participant, in the recall, or not), which differs from autobiographical events in which the scoring might be more subjective. The scoring was performed by DL with input by IM. In this experiment, novel intrusions were not considered; it is noteworthy that they were extremely rare.

List of abbreviations

AM: autobiographical memory
BPM: beats per minute
BfS: Befindlichkeits-Skala
EAM: episodic autobiographical memory
ECG: electrocardiography
EDA: electrodermal activity
EEG: electroencephalography
EM: episodic memory
fMRI: functional magnetic resonance imaging
HR: heart rate
PCA: Principal Component Analysis
RKG: Remember / Know / Guess
SCR: skin conductance response
VE: virtual environment
VR: virtual reality
